# Systemic inflammatory indices for predicting prognosis of myelofibrosis

**DOI:** 10.1038/s41598-023-39077-7

**Published:** 2023-08-02

**Authors:** Tuba Ersal, Vildan Özkocaman, İbrahim Ethem Pınar, Cumali Yalçın, Bedrettin Orhan, Ömer Candar, Sinem Çubukçu, Tuba Güllü Koca, Fazıl Çağrı Hunutlu, Şeyma Yavuz, Rıdvan Ali, Fahir Özkalemkaş

**Affiliations:** Division of Hematology, Department of Internal Medicine, Faculty of Medicine, Bursa Uludag University, 16059 Bursa, Turkey

**Keywords:** Biomarkers, Prognostic markers

## Abstract

The impact of inflammatory markers such as systemic immune-inflammation (SII) index and systemic inflammation response index (SIRI) on myelofibrosis (MF) prognosis was evaluated for the first time in this study. Data from 60 patients diagnosed with MF between March 2011 and September 2022 were retrospectively analyzed. In addition to disease-related markers, the impact of SII and SIRI on prognosis was evaluated. In our study, the overall median survival (OS) was 64 months. OS was significantly shorter in patients older than 65 years, with high ferritin and lymphocyte levels, transfusion dependence at diagnosis, platelet count below 100 × 10^9^/L, Hb level below 8 g/dl, and high risk according to the dynamic international prognostic scoring system (DIPSS)-Plus score. When these variables were included in the multivariate Cox regression model, it was found that being older than 65 years, having a high ferritin value, being at high risk according to the DIPSS-plus score and Hb values below 8 increased the risk of death. Platelet-to-lymphocyte ratio (PLR) and SII index were lower in patients with a fatal outcome. No statistically significant relationship was found between SIRI and mortality. The findings of this study showed that low PLR and high ferritin were associated with poor prognosis in MF. Elevated SII and SIRI, evaluated for the first time in patients with myelofibrosis, did not predict prognosis. Since non-inflammatory variables play a role in the pathogenesis of MF, bone marrow indicators and systemic inflammation indicators derived from hematologic parameters may not be accurate.

## Introduction

Myelofibrosis (MF) is a BCR-ABL1-negative myeloproliferative neoplasms (MPN) characterized by anemia, extramedullary hematopoiesis, bone marrow fibrosis, splenomegaly, constitutional symptoms, and acute myeloid leukemia progression^1^. Most patients carry a mutation in the *JAK-2, CALR, or MPL* genes^2^, which contributes to the JAK-2-signal-transducer-and-activator-of-transcription signaling pathway and the high inflammatory state characteristic of these diseases. Inflammation plays a crucial role in the development and progression of MPN.

The prognosis of MF varies greatly. While some patients only have a few months to survive, others live for more than 20 years. The three leading causes of death are hemorrhage, infection brought on by bone marrow loss, and transformation into acute leukemia^3^.

A good risk stratification model provides information about the prognosis of patients, affecting the decision whether the patient is included as a candidate for allogeneic stem cell transplantation and thereby the treatment. To evaluate the mortality risk of MF patients, the dynamic international prognostic scoring system (DIPSS)^4^ or DIPSS-plus is generally used^5^.

It is well established that inflammation affects all phases of tumor growth and can increase the risk of developing a tumor (triggering the first genetic mutation, tumor development, metastasis, and progression). Therefore, inflammation parameters are strong candidates for predicting cancer prognosis. Numerous inflammatory indicators have recently been linked to a poor prognosis for cancer, including C-reaction protein (CRP), neutrophil-to-lymphocyte ratio (NLR), and platelet-to-lymphocyte ratio (PLR)^6–8^. The systemic immune-inflammation (SII), which was first used in hepatocellular cancer in 2014 as a new inflammation marker based on peripheral neutrophil, platelet, and lymphocyte counts^9^ and the systemic inflammation response index (SIRI), which was developed to predict survival in patients with pancreatic cancer and based on peripheral neutrophil, monocyte, and lymphocyte counts^10^, have also been used as inflammatory biomarkers to predict prognosis in many cancer types^11–14^.

In this study, in addition to the potential prognostic markers examined in other retrospective studies to date, we investigated the prognostic value of SII and SIRI in MF for the first time in the literature.

## Materials and methods

The data of 60 patients who were followed up with the diagnosis of MF between March 2011 and September 2022 at Bursa Uludag University Hematology Department Clinic were retrospectively analyzed. All patients met the 2016 World Health Organization criteria for PMF^15^ or the 2008 international working group for myelofibrosis research and treatment (IWG-MRT) criteria for SMF^16^. Patients were divided into four groups as low, intermediate-1, intermediate-2, and high-risk groups according to DIPSS and DIPSS-plus scores. The effect of age, MF subtype, JAK-2 mutation status, erythrocyte transfusion dependence, presence of constitutional symptoms, splenomegaly, leukocyte, hemoglobin, platelet, CRP, mean corpuscular volume, mean platelet volume (MPV), red blood cell distribution width (RDW), LDH, ferritin levels, TS and CAR, PNI, NLR, PLR, leukocyte to lymphocyte ratio (WLR), ferritin to lymphocyte ratio (FLR), lymphocyte to LDH ratio (LLR), DIPSS and DIPSS-plus risk group, and SII and SIRI, which were examined for the first time in MF, on prognosis was investigated in all patients. SII was calculated as platelet count × neutrophil count/lymphocyte count in peripheral blood and SIRI was calculated as neutrophil count × monocyte count/lymphocyte count. Mortality was defined as patients who died during follow-up. Our study was conducted under the institutional research committee’s ethical standards and according to the 1964 Helsinki Declaration. This study was approved by the clinical research ethics committee of Bursa Uludag University Faculty of Medicine (Decision No: 2022-18/20).

### Statistical analysis

Statistical analyses were performed using IBM SPSS Statistics for Windows Version 25.0 (Statistical Package for the Social Sciences, IBM Corp., Armonk, NY, USA). Descriptive statistics were presented as n and % for categorical variables and mean ± SD, median (IQR) for continuous variables. Data were analyzed in terms of normality assumptions. For continuous variables with Kolmogorov–Smirnov values p > 0.05, independent samples t-test was used as the parametric test to evaluate the difference in mortality between the groups. Chi-square test was used to compare categorical variables. Three receiver operating characteristic (ROC) curve analysis was performed for SII and ferritin values to predict mortality. Kaplan–Meier method was used to compare survival times between various variables. Finally, multivariate Cox regression results of various clinical factors on mortality risk were presented. p < 0.05 was considered statistically significant in all analyses.

### Ethical approval

All procedures performed in studies involving human participants were in accordance with the ethical standards of the institutional and/or national research committee and with the 1964 Helsinki declaration and its later amendments or comparable ethical standards. Informed consent: Informed consent was obtained from all individual participants included in the study.

## Results

Sixty patients made up the study population, with 53.3% of the females and 46.7% of the males. Median age at diagnosis of 63 years. Most patients (76.6%) and those with splenomegaly (93.3%) had anemia at the time of diagnosis. Of these patients, 41.6% had massive splenomegaly. There were 41.6% of cases of constitutional symptoms, with weight loss being the most prevalent (61.5%) and high fever being the least prevalent (11.5%). Thrombosis was present in 16.6% of patients (n = 10). Four patients had cerebral vascular thrombosis, five had portal system thrombosis (three portal vein thrombosis, two splenic infarction), and one had deep vein thrombosis. JAK-2 mutation positivity was detected in 30 (58.8%) of 51 patients who underwent genetic screening. The rate of transformation to acute leukemia was 8.3%. Table 1 shows the distribution of the demographic and clinical findings of the patients.

**Table 1:** Demographic and clinical characteristics of the patients.

Variables	N	%
Age		
≤ 65	37	61.7
> 65	23	38.3
Gender		
Female	32	53.3
Male	28	46.7
MF subtype		
Primary MF	36	60.0
Secondary-MF-PV	14	23.3
Secondary-MF-ET	10	16.7
JAK-2 V617F mutation		
Negative	21	41.2
Positive	30	58.8
Leukocyte		
≤ 25 × 10^9^/L	51	85.0
> 25 × 10^9^/L	9	15.0
Hemoglobin		
< 10 g/dL	35	58.3
≥ 10 g/dL	25	41.7
Platelet		
< 100 × 10^9^/L	13	21.7
≥ 100 × 10^9^/L	47	78.3
Constitutive symptom		
No	35	58.3
Yes	25	41.7
ES Tx dependence		
No	38	63.3
Yes	22	36.7
DIPSS risk		
Low	8	13.3
Int-1	18	30.0
Int-2 + high	34	56.7
DIPSS-plus risk		
Low	8	13.3
Int-1	14	23.3
Int-2	31	51.7
High	7	11.7
Reticulin fibrosis		
Grade 2–3 +	5	8.3
Grade 3 +	8	13.3
Grade 3–4 +	12	20.0
Grade 4 +	35	58.3
Collagen fibrosis		
No	19	31.7
Yes	41	68.3
Leukemic transformation	5	8.3
Outcome		
Alive	24	40.0
Exitus	36	60.0

*MF* myelofibrosis, *JAK-2* Janus kinase 2, *ES* erythrocyte suspension, *Tx* transfusion, *DIPSS* Dynamic International Prognostic Scoring System.

**Figure 1:**
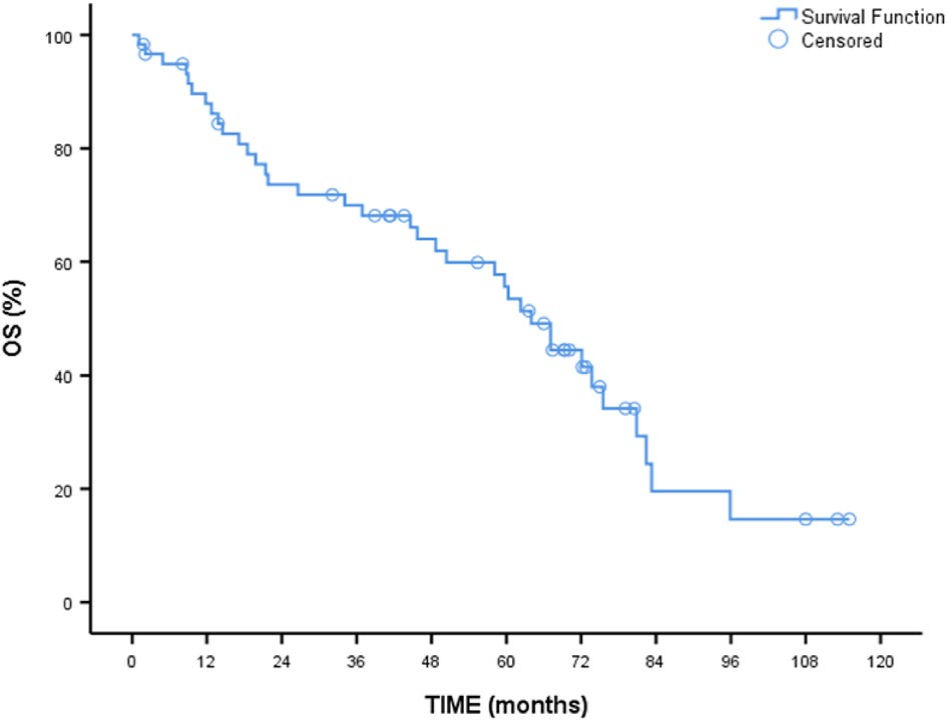
Overall survival of patients.

As seen in Table 2, median overall median survival (OS) was 64 months (95% CI 54.88–73.10). Two-year OS was 73.6%, while five-year OS was 55.6%. No difference was found between the MF subtypes (p = 0.825). Median OS was significantly different between the age groups (p = 0.005) (Fig. 1). Median survival was 73.7 months in patients under 65 years of age (95% CI 68.07–79.32) compared to 44.6 months in patients over 65 years of age (95% CI 10.66–78.53). Two-year OS and five-year OS were 83.3% and 74.1% in patients under 65 years of age compared with 56.4% and 25.6% in patients over 65 years of age.

**Table 2:** OS comparisons according to patient variables.

Overall survive (months)	Median (%95 CI)	p	HR (95%CI)	p
General	64.00 (54.88–73.10)			
Age				
≤ 65	73.70 (68.07–79.32)	**0.005**	**Ref.**	
> 65	44.60 (10.66–78.53)		2.49 (1.28–4.83)	**0.007**
Subtype				
Primary MF	64.00(49.45–78.54)	0.825	**Ref.**	
Secondary—MF-PV	58.10 (27.60–88.59)		1.25(0.57–2.72)	0.567
Secondary—MF-ET	80.90 (–)		1.20 (0.45–3.20)	0.716
JAK-2 mutation status				
Negative	64.00 (33.30–94.69)	0.806	**Ref.**	
Positive	72.10 (57.58–86.51)		0.91 (0.43–1.91)	0.806
Constitute symptom				
No	72.10 (56.68–87.51)	0.365	**Ref.**	
Yes	62.30 (3.96–120.63)		1.35 (0.69–2.63)	0.368
ES Tx dependence				
No	73.70 (58.26–89.13)	** < 0.001**	**Ref.**	
Yes	17.10 (0.00–39.79)		3.29 (1.68–6.43)	** < 0.001**
Leukocyte				
≤ 25.000 × 10^9^/L	64.00 (50.79–77.20)	0.721	0.85(0.35–2.05)	0.722
> 25.000 × 10^9^/L	21.40 (0.00–88.75)		**Ref.**	
Hemoglobin				
< 10 g/dL	44.60 (2.54–86.65)	0.168	**Ref.**	
≥ 10 g/dL	72.10 (58.87–85.32)		0.62 (0.31–1.23)	0.172
Hemoglobin				
≤ 8 g/dL	34.10 (6.13–62.07)	**0.027**	2.16 (1.07–4.36)	**0.031**
> 8 g/dL	72.10 (59.39–84.80)		**Ref.**	
Platelets				
< 100 × 10^9^/L	17.10 (6.88–27.31)	**0.002**	**Ref.**	
≥ 100 × 10^9^/L	72.10 (60.29–83.90)		3.10 (1.43–6.69)	**0.004**
DIPSS score				
Low	73.10 (53.64–93.75)	0.130	**Ref.**	
INT-1	–		0.8 (0.23–2.76)	0.732
INT-2	45.70 (28.94–62.45)		1.77 (0.61–5.15)	0.289
DIPSS plus score				
Low	73.70 (47.43–99.66)	** < 0.001**	**Ref.**	
INT-1	72.10 (–)		0.77 (0.2–2.91)	0.711
INT-2	59.70 (37.17–82.22)		1.41 (0.48–4.19)	0.527
HIGH	9.60 (8.06–11.14)		32.75 (6.66–160.95)	** < 0.001**
Reticulin fibrosis				
2–3	–	0.189	**Ref.**	
3	83.30 (25.04–141.55)		1.61 (0.31–8.35)	0.569
3–4	75.50 (–)		1.06 (0.2–5.50)	0.939
4	60.30 (53.05–67.54)		2.70 (0.61–11.87)	0.187
Kollagen fibrosis				
No	75.50 (30.25–120.75)	0.112	**Ref.**	
Yes	62.30 (54.65–69.95)		1.86 (0.85–4.07)	0.117
Transferrin saturation				
≤ %20	60.30 (44.53–76.06)	0.880	**Ref.**	
> %20	68.88 (59.60–84.59)		0.94 (0.45–1.94)	0.880

Significant values are in bold.

Kaplan–Meier curve, Long rank test, Univarite Cox Regression Test, p < 0.05 statistically significant.

*MF* myelofibrosis, *PV* polystemia vera, *ET* essential thrombocytosis, *JAK-2* Janus kinase 2, *ES* erythrocyte suspension, *Tx* transfusion, *DIPSS* dynamic international prognostic scoring system, *INT-1* intermediate-1, *INT-2* intermediate-2.

A significant difference was found in OS with respect to DIPSS-plus risk groups (p < 0.001). Median survival was 73.7 months (95% CI 47.43–99.66) in the low-risk group, 72.1 months (95% CI –) in the intermediate-1 risk group, 59.7 months (95% CI 31.17–82.22) in the intermediate-2 risk group, and 9.6 months in the high-risk group (95% CI 8.06–11.4). A statistically significant difference was also found in median OS between the high-risk group and all other risk groups (p < 0.001). Two-year OS and five-year OS were 100% and 83.3% in the low-risk group, compared with 92.3% and 84.6% in the intermediate-1 risk group. In the intermediate-2 risk group, two-year OS was 76.3% and five-year OS was 49.9%. In the high-risk group, all patients died within two years.

PLR (p = 0.048), SII (p = 0.018) and lymphocyte count (p = 0.033) showed a statistically significant difference between patients with and without mortality. PLR and SII were lower in patients with mortality compared to patients without mortality, while ferritin and lymphocyte levels were higher. No significant difference was found in survival with respect to other variables (neutrophils, platelets, NLR, LLR, WLR, FLR, RDW, MPV, CRP, LDH, spleen size, serum iron, iron binding capacity, TS, PNI, CAR).

A significant relationship was found between OS and Hb levels below 8 g/dL (p = 0.027), transfusion dependency (p < 0.001), and platelet count below 100 × 10^9^/L (p = 0.002), while no statistically significant relationship was found between OS and MF subtypes, positive or negative JAK-2 mutation status, presence of constitutional symptoms, TS < 20%, Hb < 10 g/dL, leukocyte count > 25 × 10^9^/L, degree of collagen and reticulin fibrosis, and DIPSS risk groups.

Univariate analysis results showed that age, ferritin, transfusion dependency, platelet count, DIPSS-plus risk group, Hb, and SII index variables were statistically significant for mortality risk (p < 0.05). The variables that were significant in univariate analyses were included in the multivariate Cox regression model. According to the results of the multivariate Cox regression model, it was found that being over 65 years of age (HR 7.29; 95% CI 2.44–21.75; p < 0.001), increased ferritin values (HR 1.00; 95% CI 1.00–1.01 p = 0.002), high-risk DIPSS-plus (HR 12.63; 95% CI 1.30–122.30 p = 0.029), and hemoglobin values below 8 increased mortality risk (OR 0.32; 95% CI 0.11–0.94 p = 0.038) (p < 0.001, − 2 loglikelihood = 158,326) (Table 3).

**Table 3:** Multivariate Cox regression results for various clinical variables.

Multivariate		
Variables	HR (95%CI)	p
Age (ref: ≤ 65)	7.29 (2.44–21.75)	**< 0.001**
Ferritin	1.00 (1.00–1.01)	**0.002**
ES Tx dependence (ref: no)	1.54 (0.48–4.97)	0.464
PLT (ref: < 100.000)	0.78 (0.27–2.29)	0.663
Diagnosis DIPSS-plus risk (ref: low)		
INT-1	0.36 (0.07–1.48)	0.151
INT-2	0.64 (0.14–2.88)	0.570
High	12.63 (1.30–122.30)	**0.029**
Hemoglobin (ref: < 8)	0.32 (0.11–0.94)	**0.038**
SII index	1.00 (1.00–1.00)	0.154

Significant values are in bold.

p < 0.001; − 2 Log Likelihood = 158,326.

**Figure 2:**
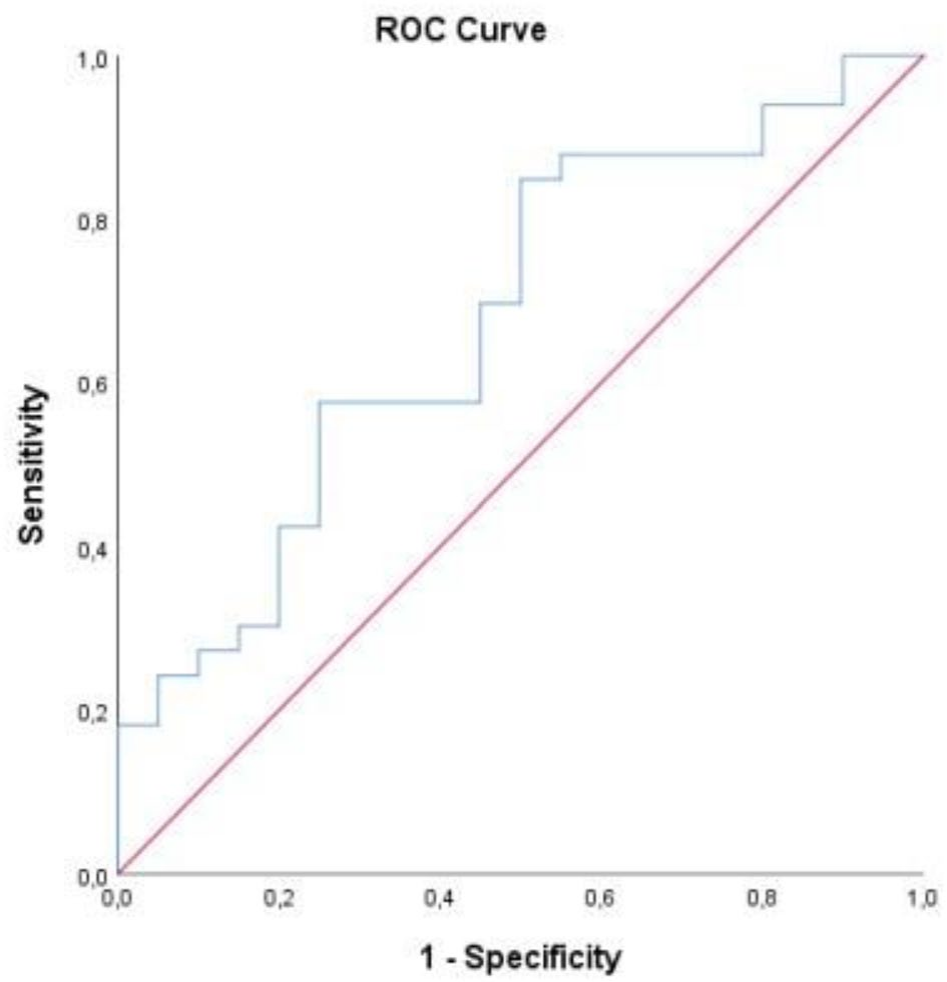
ROC curve of SII levels for predicting mortality.

**Figure 3:**
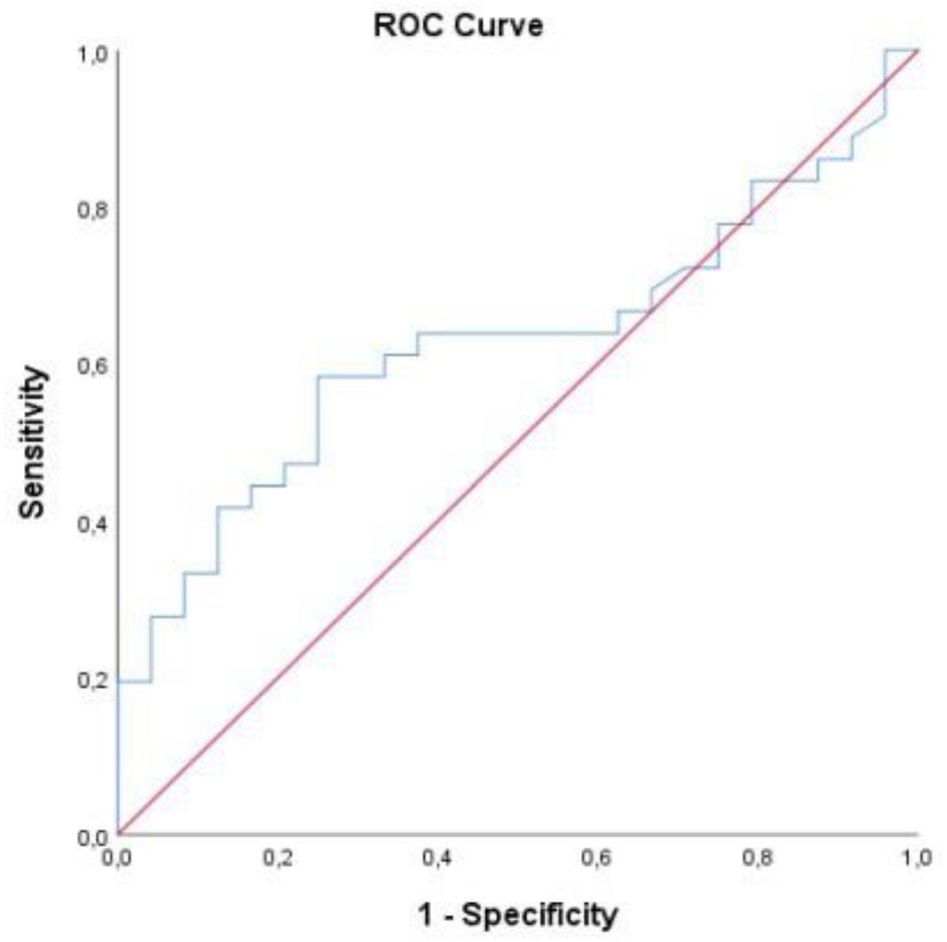
ROC curve of Ferritin levels for predicting mortality.

The predictive power of SII for mortality was statistically significant (p = 0.032). In the ROC analysis conducted to evaluate SII in predicting mortality, the area under the curve was 0.677 (95% CI 0.528–0.827) and the cut-off value for SII was 1246.78 (Fig. 2). For this value and below, sensitivity was 57.6% and specificity was 55.5%. The predictive power of ferritin was not statistically significant for mortality (p = 0.097) (Table 4, Fig. 3).

**Table 4:** Predictive power of SII and Ferritin in differentiating mortality.

	AUC	95% CI	Cut-off	Sensitivity (%)	Specificity (%)	p
SII	**0.677**	**0.528–0.827**	** ≤ 1246.78**	**57.6**	**55.0**	**0.032**
Ferritin	0.627	0.487–0.768	≥ 148.50	63.9	62.5	0.097

Significant values are in bold.

*AUC* Area under the curve, *95%CI* Confidence interval.

## Discussion

Accurate risk assessment is crucial for developing the best treatment strategy in MF, which is one of the BCR-ABL-negative MPNs accepted as a model of inflammation-related cancer development, especially in young patients. Widely accepted scoring systems require genetic evaluation and tests may be difficult to access in some centers. The current study assessed prognostic indicators in patients with MF. To the best of our knowledge, this is the first study to have examined the relationship between SII and SIRI and mortality in MF patients in the literature. Inflammatory indicators and parameters that could affect prognosis were assessed in 60 patients.

Indices such as SII and SIRI are thought to be associated with the prognosis of various tumors. A meta-analysis by Yang et al. evaluating 22 studies including 7657 patients revealed that high SII was clearly associated with lower OS, time to recurrence, progression-free survival, cancer-specific survival, relapse-free survival, and disease-free survival^17^. These results suggest that high SII may be a potential prognostic marker in patients with various cancers and may be associated with poor overall outcomes. A study by Geng et al. in patients with esophageal cancer showed that median OS was significantly higher in patients with low SIRI^18^.

In this study, the relationship between MF and SII was evaluated for the first time in the literature; and paradoxically, mortality was found to be lower in patients with MF compared to patients without MF (p = 0.018). This discrepancy results from patients with a fatal course having higher lymphocyte numbers and lower platelet counts. SII lost its relevance when these variables were incorporated into the multivariate Cox regression model. Additionally, SIRI was also examined for the first time in MF patients and found not to be associated with mortality (p = 0.492).

Anemia is a disease characteristic most consistently associated with poor prognosis in MF^5,19–21^. The most commonly used threshold in prognostic models is 10 g/dL. Transfusion dependence has had poor prognostic significance in MF^22–24^. There is ongoing debate about the relationship between transfusion dependence and poor prognosis in chronic MPN. Some authors argue that transfusion dependence affects survival through the adverse effects of chronic erythrocyte transfusion, such as iron overload and transfusion-related immunomodulation. In the present study, when the Hb cut-off point was taken as 10 g/dL, no difference was found between the groups in terms of survival (p = 0.168). However, when the cut-off point was taken as 8 g/dL, a significant difference was found in median OS (p < 0.027). Hb level ≤ 8 g/dL was determined as a marker of poor prognosis. When included in the multivariate Cox regression model, Hb < 8 g/dL increases the risk of mortality (HR 0.32; 95% CI 0.11–0.94 p = 0.038).

Some studies have found that thrombocytopenia was associated with poor prognosis^5,20,21,25^, but it was noted that low platelet counts are frequently associated with anemia and collinearity in multivariate regression models may make it difficult to characterize thrombocytopenia as an independent prognostic factor^19^. In the present study, platelet count was lower in patients with a mortal course, but the difference was not statistically significant (p = 0.085). When the cut-off value for platelet count was taken as 100 × 10^9^/L, a significant difference was found in median OS (p = 0.002). Median OS was significantly shorter in patients with platelet count below 100 × 10^9^/L (72.1 months versus 17.1 months).

The transfusion dependency at diagnosis or during MF is an indicator of poor prognosis^22,23^. In our patients, median OS was 73.7 months in the group without transfusion dependence and 17.1 months in the group with transfusion dependence, which was significantly lower (p < 0.001).

Consistent with previous studies, age was associated with OS in both univariate analysis and multivariate Cox regression analysis. When the cut-off point for age was taken as 65 years, a significant difference was found in OS between the groups (p = 0.005). When included in the multivariate Cox regression model, it was found that mortality risk was significantly higher in those older than 65 years (HR 7.29; 95% CI 2.44–21.75; p < 0.001).

Currently, the most widely used prognostic scoring system in MF is DIPSS-plus. DIPSS-plus was used in 967 consecutive patients at the Mayo Clinic and resulted in median survival of 1.8, 3.6, 7.8, and 17.5 years for high, intermediate-2, intermediate-1, and low-risk patients, respectively^26^. When the patients in this study were divided into risk groups according to DIPSS-plus, a statistically significant difference was found between the median OS times (p < 0.001). Median OS was 73.7 months in the low-risk group, 72.1 months in the intermediate-1 risk group, 59.7 months in the intermediate-2 risk group, whereas it was 9.6 months in the high-risk group (p < 0.001). In the multivariate Cox regression analysis, a significant difference was found between the low-risk group and the high-risk group persisted (HR 12.63; 95% CI 1.30–122.30 p = 0.029), while the significance between the low-risk group and intermediate-1 and intermediate-2 risk groups disappeared (p = 0.151, p = 0.570, respectively).

With respect to iron metabolism, studies have shown that high ferritin value and low TS are associated with low OS in MF. Lucijanic et al. evaluated the prognostic impact of low TS in 87 patients with PMF. Low TS was found to have a detrimental effect on the survival of PMF patients, independent of anemia and ferritin levels^27^. In the present study, ferritin level was found to be higher in patients with mortality (p = 0.024) and when included in the multivariate Cox regression model, it was found that an increase in ferritin levels increased the risk of mortality (HR 1.00; 95% CI 1.00–1.01 p = 0.002). However, there was no statistically significant relationship between TS, serum iron level, iron binding capacity, RDW, and mortality. Numerous studies have been published in the literature showing the link between several inflammation markers, including NLR and PLR, and a poor prognosis for cancer^7,8,28^. In a study evaluating NLR and PLR in MF, these values were found to be significantly higher in patients compared to healthy controls. In univariate analyses, shorter overall survival was observed in patients presenting with high NLR and low PLR^29^. In the same study, increased RDW was associated with survival (p = 0.039). In MF, high CRP is associated with features of more advanced disease and a trend toward worse clinical outcomes as part of individual parameters or different prognostic scores^30–32^. In this study, no correlation was found between CRP and NLR and survival. Consistent with the literature, survival was shorter in patients with low PLR values (p = 0.048).

CAR has recently been recognized as an inflammatory biomarker and prognostic factor in several malignant neoplasms^33,34^. However, a study evaluating CAR in patients with MF reported that higher CAR was associated with lower OS^35^. PNI is an index reflecting a patient’s inflammatory, nutritional, and immune status. In a study evaluating PNI in MF patients, low PNI predicted worse survival independent of DIPSS^36^. In the present study, however, CAR and PNI had no effect on survival.

The mean LDH level was higher (p = 0.108) and spleen size was larger (p = 0.122) in patients with a mortal outcome, but this was not statistically significant.

This study has certain limitations. The study was conducted retrospectively and in a single-center.

In conclusion, the results obtained in this study show that elevated SII and SIRI, which have prognostic significance for many cancers, cannot be used as markers for poor prognosis in MF. Since the pathology of MF directly involves the bone marrow unlike solid organ cancers, these inflammation markers may be insufficient to predict prognosis. Further clinical studies are needed to confirm these results.

## Data Availability

The datasets used and/or analysed during the current study available from the corresponding author on reasonable request.
